# Molecular Modeling and Simulation Tools in the Development of Peptide-Based Biosensors for Mycotoxin Detection: Example of Ochratoxin

**DOI:** 10.3390/toxins9120395

**Published:** 2017-12-06

**Authors:** Aby A. Thyparambil, Ingrid Bazin, Anthony Guiseppi-Elie

**Affiliations:** 1 Center for Bioelectronics, Biosensors and Biochips (C3B), Texas A&M University, College Station, TX 77843, USA; athypar@tamu.edu; 2 Department of Biomedical Engineering, College of Engineering, Texas A&M University, College Station, TX 77843, USA; 3 Laboratoire de Génie de l’Environnement Industriel( LGEI), Institut Mines Telecom (IMT) Mines Ales, University of Montpellier, 30100 Ales, France; ingrid.bazin@mines-ales.fr; 4 ABTECH Scientific, Inc., Biotechnology Research Park, 800 East Leigh Street, Richmond, VA 23219, USA

**Keywords:** peptides, mycotoxins, ochratoxin, biosensors, all-atom molecular dynamics, molecular recognition NFO4, BEMD, MSM

## Abstract

Mycotoxin contamination of food and feed is now ubiquitous. Exposures to mycotoxin via contact or ingestion can potentially induce adverse health outcomes. Affordable mycotoxin-monitoring systems are highly desired but are limited by (a) the reliance on technically challenging and costly molecular recognition by immuno-capture technologies; and (b) the lack of predictive tools for directing the optimization of alternative molecular recognition modalities. Our group has been exploring the development of ochratoxin detection and monitoring systems using the peptide NFO4 as the molecular recognition receptor in fluorescence, electrochemical and multimodal biosensors. Using ochratoxin as the model mycotoxin, we share our perspective on addressing the technical challenges involved in biosensor fabrication, namely: (a) peptide receptor design; and (b) performance evaluation. Subsequently, the scope and utility of molecular modeling and simulation (MMS) approaches to address the above challenges are described. Informed and enabled by phage display, the subsequent application of MMS approaches can rationally guide subsequent biomolecular engineering of peptide receptors, including bioconjugation and bioimmobilization approaches to be used in the fabrication of peptide biosensors. MMS approaches thus have the potential to reduce biosensor development cost, extend product life cycle, and facilitate multi-analyte detection of mycotoxins, each of which positively contributes to the overall affordability of mycotoxin biosensor monitoring systems.

## 1. Introduction

**Figure 1:**
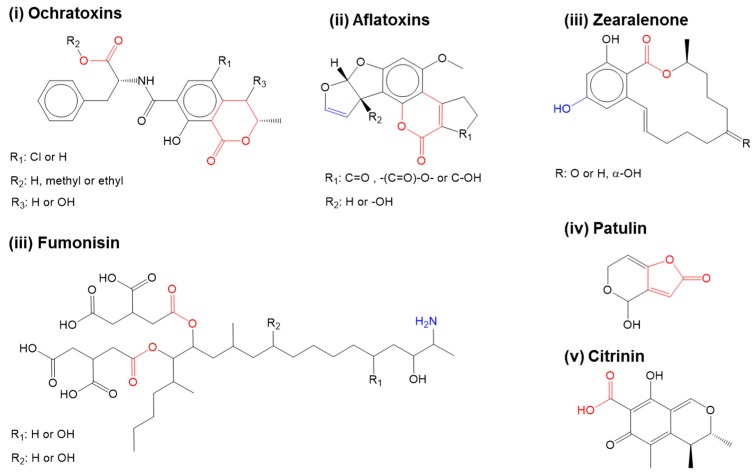
Mycotoxins commonly found in food. Adapted from references [5]. Reproduced from [11], Copyright Springer, 2014. Carboxyl derivatives within the ester bonds and lactone side groups which often play a role in toxicity (red). Other specific groups responsible for mycotoxin toxicity are represented in blue.

Over the past 2–3 decades, increasing globalization has drastically improved the access to food and feed beyond one’s geographical boundaries. The lengthening of food-feed supply chains from the farm to the plate have also heightened the concerns on food-feed-safety because of its vulnerability to potential risks in each stage of food-feed production [1,2,3]. Factors such as the climatic shifts in different geographical locations, the use of unsafe water in food cleaning and processing, and the lack of appropriate food-feed storage infrastructure can all stimulate the growth of storage fungi such as *Aspergillus ochraceus* and *Penicillium verrucosum* and so alter risk associated with food-feed safety by mycotoxin contamination [4,5]. Figure 1 shows the chemical structure of the mycotoxins commonly found in food and feed [4,5]. Many of these mycotoxins tend to co-occur with other sometimes structurally un-related mycotoxins. Persistent low-grade exposure to such mycotoxins via transdermal and oral routes can lead to allergic and hypersensitive reactions, endocrine disruption, and increased cancer risks [4,5,6,7,8,9]. In extreme cases, such mycotoxin overexposure could even be life-threatening [4,5,6,7,8,9]. In addition, co-exposures to multiple mycotoxins are known to have multiplicative and synergistic effects on human and animal health. With such increasing risk of food safety from mycotoxin contamination, it has become more important to monitor and regulate the content and extent of mycotoxin contamination in food and feed products via analytical systems such as biosensors [1,4,10,11].

**Figure 2:**
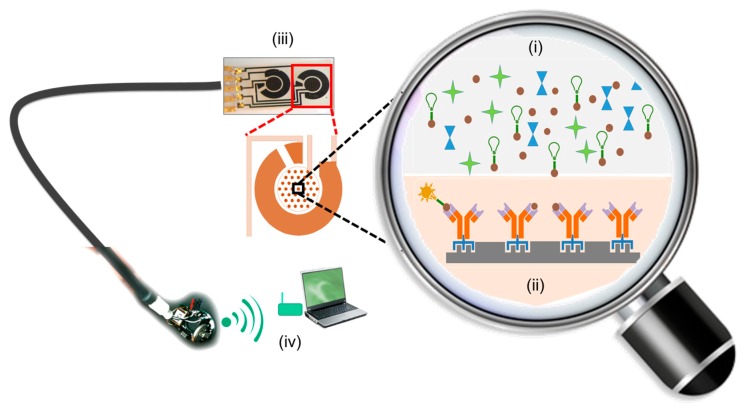
Schematic of a mycotoxin monitoring biosensor system relying on a competitive binding assay. The sample matrix with the mycotoxin of interest, mycotoxin tagged with the reporter tag (shown as a green bulb), along with other potential interfering agents are introduced in (**i**). The biotransducer comprising the biorecognition receptor (**ii**) was chemically modified to incorporate a linker. The linker facilitates the immobilization of the biorecognition receptor to the solid transducer surface. As most binding events cannot be directly monitored, change in the physical properties of the reporter tags (green → yellow conversion) are indicative of binding events between the biorecognition receptor and the target analyte. The stoichiometric ratios of the untagged mycotoxins and mycotoxins tagged with the reporter tag are then indicative of the mycotoxin content in the sample matrix. Shifts in the physical properties of reporter tag from multiple sensor arrays are integrated and processed by the transducers (**iii**) and transmitted as meaningful data, that could be visualized as a digital readout on a smart phone or laptop (**iv**).

The general schematic of a biosensor system highlighting the different components involved in mycotoxin detection is shown in Figure 2. In principle, any biomolecular entity that is capable of recognizing a target mycotoxin can be used as a bioreceptor (Figure 2ii). During the last decade, the gold standard for monitoring the mycotoxin content in a variety of food matrices has involved molecular recognition via immuno-chemical methods [12,13]. Such immunochemical detection methods can vary from a simple immunoassay to highly sophisticated immune-biosensor. The diversity in these types of assays, the underlying mechanisms of recognition and the variations in formats have all been extensively reviewed [12,13]. Nevertheless, the core principle in these immunoassays rely on the affinity of monoclonal antibodies (Mab) to specifically bind to target mycotoxins. The affinity of antibodies to specific mycotoxins is especially more important when the mycotoxin concentrations in the food or feed matrices are very low (~nM). Since mycotoxins are low-molecular-weight haptens, mycotoxin-specific antibody production is not easy, being costly and highly laborious. Additionally, since the ordered structure of antibodies is critical for its function, matrix effects as well as many of the environmental variables involved in day-to day operational processes (like buffers, salts, temperature, inhibitors, storage conditions and others) can limit the service-life of these bio-receptors [14]. Maintaining the operational stability of the antibody-based molecular recognition bioreceptor under such operating conditions adds to the operational cost and relegates the fabrication process to educated trial-and-error approaches (and are therefore less likely to be optimized) [15]. Despite these difficulties, antibodies to most mycotoxins are commercially available and widely used in immunoassays. However, the limited affordability of antibody-based sensing elements have affected the self-hapten monitoring/regulatory capacity of the producers and regulatory groups from low-income economies, risking the introduction of mycotoxins into the food and feed supply chains [2,16,17]. The high variability in the mycotoxin contamination across different origin points in the supply chain require increased awareness and ongoing surveillance for mycotoxins [4]. By enabling low-income groups with the access to a more affordable mycotoxin monitoring device would not only help in complying with the regulations but also provide access to a wider range of export markets with diverse regulatory requirements, which in the longer run would help reduce food loss and reduce global hunger, strengthen local economies, and safeguard human and animal health. Concerned producers and regulatory bodies are therefore seeking newer, faster, more convenient and cost-efficient methods of real-time detection and monitoring of mycotoxins in a variety of food matrices.

**Figure 3:**
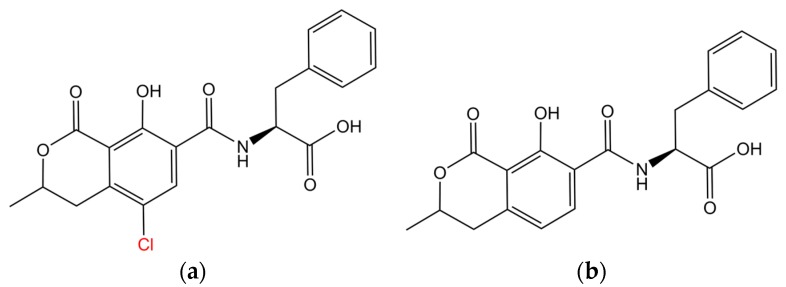
Chemical structure of (**a**) ochratoxin-A (OTA) and (**b**) ochratoxin-B (OTB). The structural analogues differ in ‘Cl’ moiety, which is highlighted in red.

Over the past few decades, the Bioelectronics, Biosensors and Biochips (C3B^®^) group has been engineering a wide range bioanalytical microsystems in the service of human health and medicine [18,19,20,21,22,23,24,25,26,27]. Recently, our group has also been involved in the development of peptide-based optical and electrochemical biosensors for continual OTA monitoring [28,29,30,31,32]. Peptides can be chemically synthesized to virtually any mycotoxin target. Compounded with the robustness and operational stability in a wide range of buffer solutions, matrices, and amenability to targeted modifications for detection or immobilization purposes, biosensing recognition receptors based on peptides are often considered more cost-effective than antibody-based sensing elements [33]. Over the last decade, a variety of platforms based on immobilized peptides have been developed against a variety of mycotoxins with varying levels of success [14,34,35,36,37,38]. For example, consider the case of ochratoxins [6]. Though at least three different structural variants of ochratoxin are known to naturally occur, only ochratoxin A (OTA) and its non-chlorinated analogue, ochratoxin B (OTB) are found prevalently in food and feed. Structures of OTA and OTB are shown in Figure 3. Of these two ochratoxins, OTA is more toxic and a variety of biosensing elements and detection formats have been developed against OTA during the last decade (Table 1). As evident, though some of these assay formats have reported to detect sub-regulatory levels of OTA in a wide range of food and environmental matrices, the performance of peptide-based molecular assemblies are yet to surpass the performances of the equivalent monoclonal or polyclonal or recombinant antibody-based formats (Table 1).

In addition to improving the mycotoxin binding properties of the peptide receptors, the grand challenge in the fabrication of any biosensor for mycotoxin monitoring lies in the ability to predict and control the mycotoxin-binding properties of the molecular recognition receptor of the biotransducer. While it is often assumed that the mycotoxin binding behavior of a peptide *in-solution* is replicated even when the peptide is chemically modified for immobilization, chemically conjugated for labeling and/or physicochemically immobilized on a solid support, this assumption may not necessarily be true [28,29,30,31,32]. Many studies have in fact shown that a variety of factors in the synthesis of the molecular recognition receptor, in the fabrication of the biotransducer using that receptor, and in the operational processes associated with the use of that biotransducer can promote, inhibit or have no effect on the *in-solution* affinities and selectivity of the biological recognition molecule [29,31,45,48]. Among these operational parameters are pH, temperature, chemistry and topography of device surface, recognition molecule density, site of chemical labels/tags as well as other effects. Our experiences have shown the traditional experimentation, especially one-variable at a time approaches, may not be sufficient to address the design and optimization challenges involved in the fabrication of biosensing receptors for optimized hapten recognition. Also, such approaches may not necessarily capture the molecular-details necessary to optimize the bioreceptor within the biosensor. It is in this context that molecular modeling and simulation (MMS) tools are highly relevant in providing molecular-level insights that could aid the design and optimization of the molecular recognition receptors for mycotoxin detection and monitoring. MMS tools aid the representation of geometries (bond lengths, bond angles, torsion angles), energies (e.g., heat of formation, activation energy), and many other physicochemical properties of the peptide and mycotoxins. It is also possible to simulate the molecular behavior of the peptide and mycotoxins using either Newton’s equations of motion or quantum equations. In this context, peptides, being relatively small molecules, are tractably addressed using MMS, while antibody fragments and whole antibodies, being quite large molecules, are computationally quite expensive to simulate. The incorporation of MMS approaches into the mainstream of biosensor design and fabrication could potentially minimize the product development lifecycle and provide a rational approach to optimize device performance. While such a synergistic approach has shown success in medicinal chemistry and chemical toxicology, its adoption and success rate has been minimal in the biosensor field, especially for monitoring mycotoxins. In this article, our objectives were firstly, to outline the specific experimental challenges encountered in each stage of the preparation of the molecular recognition receptor and the fabrication of the biotransducer. Secondly, our rationale for the use of MMS tools to address these challenges are outlined. Though much of the discussion will be limited to the current or ongoing work on OTA, the general scope of the strategy outlined in the current article is scalable to any peptide-based molecular assemblies used for mycotoxin detection.

## 2. Challenges in the Fabrication of Sensing Element for Mycotoxin Monitoring

In this section, challenges faced in the identification, biomolecular design and engineering, and fabrication of molecular recognition receptors will be discussed under two broad categories: (1) screening for peptide receptors with mycotoxin specificity; and (2) sub-molecular processes influencing the performance of molecular recognition receptors.

### 2.1. Screening of Peptide Receptors for Mycotoxin Specificity in Monitoring

**Figure 4:**
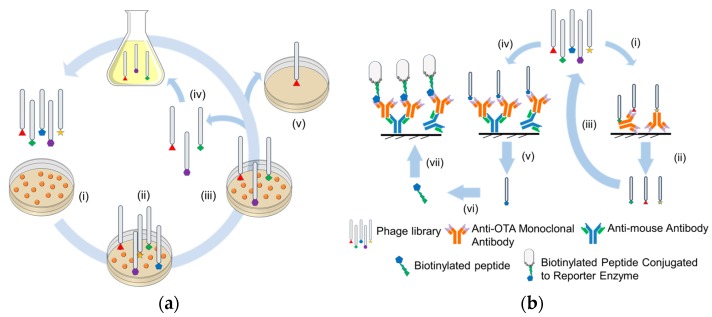
Schematic of the (**a**) phage library and (**b**) mimotope mapping approaches for generating peptide receptors for OTA capture. Phage library approach involves coating the immunoplate with haptens (**ai**) and then biopanning with the phage library of interest (**aii**). The non-specifically bound and weakly bound phages are washed off in the initial step and the more strongly bound phages are held onto the immunoplate (**aiii**). The more strongly bound phages are desorbed off the surface and may be reverse transcribed or enriched in bacterial/vector culture (**aiv**). The highly selective peptide receptors are eventually screened and sequenced (**av**). Mimotope mapping approach involves two approaches of biopanning the peptide library as demonstrated by (**bi**–**biii**) and (**biv**–**bvii**). In the first approach, the anti-OTA monoclonal antibody was directly coated on the surface and biopanned with the peptide library (**bi**). Peptides with higher non-specificity were either screened (**bii**) or were enriched by reverse transcription in bacterial/vector culture to produce newer generation of peptide libraries (**biii**) that could be further screened for OTA binding. In the second approach, peptides were biopanned from a more oriented layer of anti-OTA monoclonal antibody, (**biv**,**bv**). The isolated peptides were then chemically/biologically modified (**bvi**) and the performance assessed using an indirect sandwich assay (**bvii**). Reproduced from [14]. Copyright Elsevier 2017, and [44], Copyright Elsevier 2016.

The relative placement and the number of R-groups of the amino acid residues constituting the peptide sequence determine a peptide’s affinity and selectivity to a target mycotoxin. Even a minor variation in the sequence can affect a peptide’s affinity and selectivity to a target mycotoxin. The majority of the high-affinity peptide receptors [generally a septamer (7aa) or a dodecamer (12aa)] for mycotoxin monitoring have been identified by two main combinatorial chemistry approaches: (1) phage display library technique; and (2) mimotype mapping [14]. Both approaches rely on the affinity of the peptide sequence expressed on the surface of the viral capsid of bacteriophages such as M13 (Figure 4). The M13 bacteriophage is genetically altered to express diverse peptide sequences on its viral capsid. In the phage display library technique, the phage libraries were incubated with OTA in an immunoplate to which the virus bind with varying affinity (Figure 4a). Peptides with weaker affinity to OTA are removed in the initial panning process and the successive rounds of panning were carried out to enrich peptide binders and to ensure the isolation of peptide fragments with the strongest binding affinities to the hapten. Giraudi et al. were the first to report a hexamer (SNLHPK) for OTA targeting via an evolutionary combinatorial approach, based on the selection of the best sequence extracted from a starting dipeptide library [46]. However, the intrinsic binding affinities (*K_D_* ~ 29.4 μM) of these hexamers were fairly weak and could be attributed to the limited diversity in the phage display peptide libraries that were randomly synthesized by the bacteriophage. To address this limitation, the mapping of the mimotope, a peptide which mimics the structure of an epitope, was more widely adopted in the future studies. Mimotope mapping involves peptide isolation from phage-display library by selectively screening the peptide receptor that structurally complements the hapten structure or the binding site within the hapten-specific receptor (Figure 4b). In principle, the mimotope approach should result in peptides with higher affinity to OTA than the phage display library approaches, especially when the mimotopes are derived from the specific antigen recognition sites within an antibody. Using such an approach, Liu et al. identified a peptide motif (IR(V)PMV(L)XX) from the anti-OTA monoclonal antibodies using the approach in Figure 4(bi) [49]. In their study, the peptide IRPMVDP was found to have a high affinity (*K_D_* ~ 1.7 nM). In another study, Bazin et al. designed and chemically synthesized a dodecamer peptide, NFO4 (VYMNRKYYKCCK) that would mimic the interaction of molecules containing ester (R1COOR2), and amide (R1CONHR2R) and phenol moieties with specific regions of human NADH-FMN oxidoreductase [32,47]. As expected, the intrinsic affinity of NFO4 peptides (*K_D_* ~ 79 nM) was lower than that of the mimotopes from antibodies. He et al. further improved the peptide receptor for OTA recognition in monitoring by identifying a dodecamer (AETYGFQLHAMK, *K_D_* ~ 0.13 nM) from a second-generation peptide library (Figure 4(biii)) [50]. In their study, OTA mimotopes were initially selected from random peptide libraries to identify a motif (GFQLH) sequence. On the basis of the motif, a second-generation peptide library was then constructed and mimotopes with various affinities were screened. Nevertheless, majority of the mimotopes that were identified were based on coating OTA as antigens in immunoassay via the covalent coupling of amino or carboxyl groups of the peptide and the carrier protein by using an active ester or by applying the glutaraldehyde crosslinking method. Zou et al. speculate that such antibody immobilization process could potentially block the active site of the mimotopes and instead used an indirect sandwich-type assay to generate a more oriented layer of the antibody (Figure 4(biv–bvii)) [44]. Using this approach, a biotynated septamer (GMVQTIF, *K_D_* ~ 0.07 nM) with a pentamer spacer (GGGSK) was developed as a peptide receptor for OTA. The septamer generated from the phage display library mimicked the OTA binding site within anti-OTA monoclonal antibody 2A11.

One of the main challenges with the combinatorial approach pertains to identifying peptides with optimal affinity and selectivity to OTA. The type of phage library selected restricts sequence variations in the peptide. Also, even minor sequence variations can affect a peptide’s affinity and selectivity to a target hapten. For a dodecamer peptide that is composed of twenty canonical amino acids, the number of peptides generated based on the diversity in functional group compositions (hydrophobic, hydrophilic, aromatic, positively and negatively charged) and their arrangement within the sequence could be in the order of millions—a prohibitively large amount of peptides to synthesize. Consequently, discovery of peptides with optimal activity via combinatorial approaches is a time-consuming process that involves complex laboratory procedures, with the complexity of the screening increasing exponentially with the number of executed cycles. There are several strategies that have been adopted to optimize peptides with improved affinity and selectivity to OTA such as pre-selecting a restricted library of amino-acids or limiting the number of variable positions. One such approach involves the use of high-throughput screening to filter down a large library of peptides into a shortlist of active or “*lead-like*” compounds which can be used as the basis for peptide optimization [51]. According to some estimates, high-throughput screening could currently screen up to 100,000 compounds a day, using extremely tiny volumes of reactants, at a low cost per molecule screened [52]. However, the equipment itself is expensive, usually found in industry, with a few academic exceptions.

Recently, peptide receptors for OTA binding have also been identified by a molecular modeling approach involving an incremental construction (IC) approach [39]. The incremental construction is a virtual screening approach that relies on the interaction energies between select amino acids and a target moiety within the target mycotoxins, based on which the overall affinity of the peptides are scored and ranked. The peptide’s affinity towards a target mycotoxin could be further increased either by the selective increase of its length or mutation of the residues. The obvious benefit of the IC approach lies in the ease with which the selection pool and the basic validation of the binding behavior of peptide receptors could be narrowed down to a few experimental leads. If properly applied, IC could prove to be a much valuable and a more welcome alternative to the time-consuming and cumbersome combinatorial approaches. Unfortunately, the peptide receptors identified via IC approaches are relatively inefficient than those identified via combinatorial chemistry approaches (Table 2). The relative success of IC approaches relies on the choice of benchmark data and its prospective utility, as the chemistry and the scoring functions are based on the empirical results of drug binding in physiological environment [53]. However, the working environments for mycotoxin analysis involve solvent additives such as methanol or ethanol (e.g., wine), that are less polar than water and tend to form linear H-bond with the peptide. Such change in the solvent environment may necessitate better understanding of the chemistry involved in the mycotoxin binding to the peptide and re-parameterization of the scoring functions [13]. Routine biophysical techniques such as equilibration dialysis, enzyme linked immunosorbent assay (ELISA) or surface plasmon resonance (SPR), however, lack the resolution of sub-molecular events. Additionally, the data on the affinities and selectivity of the peptide to a target mycotoxin is further complicated by the experimental conditions unique to each biophysical tools such as peptide conjugation with a larger enzyme, bio-immobilization, or chemical modification. Instead, a standardized platform capable of providing molecular level insight into the mycotoxin recognition process may make it possible to accurately compare the molecular recognition capabilities of different peptides that were identified by different research groups. Also, such standardized platforms may prove more beneficial in identifying the target libraries with the chemistry of interest and could achieve better ‘*enrichment*’ of the peptide hits.

### 2.2. Sub-Molecular Processes Influencing the Performance of Peptide Bioceceptors

The core of a biosensor system is the biotransducer, and the key component of the biotransducer is the molecular recognition bioreceptor. The overall performance of a biosensor system is directly influenced by the mycotoxin-binding properties of the molecular recognition bioreceptor. The design and fabrication of the biotransducer involves the immobilization of the peptides receptor on the insoluble phases of physicochemical transducers such as metallic electrodes, composites, ceramics, and polymers in a plurality of formats such as polymer foams, polymer membranes, hydrogels, nanomaterials, and films [54,55,56]. A generic approach to peptide immobilization involve support choices that are relatively cheap and easy to obtain, but provides a high surface-to-volume ratio to facilitate a dense packing of the peptides. Furthermore, immobilization also aids in the cost-effective usage of the molecular recognition receptors, by facilitating the recovery, stability, and possible reusability of the detection platform from the bioanalyte matrix. Higher packing densities are also essential to maximize the mycotoxin extraction from the food and/or feed matrix of interest and retaining the configurational freedom responsible for the *in solution* mycotoxin binding properties of the peptide [32]. Following the substrate selection, several strategies to immobilize peptides on a variety of materials are available and have been previously reviewed [57]. Surface immobilization of peptides are often achieved via: (1) physical adsorption; (2) covalent attachment via chemical activation of functional groups on the surface and functional groups on the peptide; (3) molecular entrapment approaches; or (4) linkers-like affinity tags such as polyhistidine-tag (6-His), biotinylation of peptide receptors and their subsequent immobilization via streptavidin-coated supports, and Watson-Crick paired DNA-directed immobilization [12,14,35,58,59]. Ideally, the support substrates onto which the peptides are immobilized should minimally interact with the peptide, should orient the binding site within the peptide towards the analyte without any steric hindrance, and not interfere with the peptide’s mycotoxin binding properties. Yet, in a recent study by Giovannoli et al., the performance of peptide-based solid phase in extracting OTA from spiked wine samples were shown to be either better, worse or relatively similar to the *in-solution* properties of the peptide depending on the differences in chemical and physical characteristics of the solid supports that were used to immobilize the peptides [45,60,61]. In addition to the surface, mycotoxin-binding properties of the molecular recognition receptor may also be affected by the process of chemical or biological modification. Peptide receptors or mycotoxins could be modified to incorporate reporter labels. The incorporation of reporter label in the peptide is important for providing a temporal visualization and quantitative measurement of the binding events involving the peptide and mycotoxin. However, depending on the type and the sites of such modifications, the mycotoxin-binding properties of the peptide receptor may be further impacted [29]. In the following sections, the sub-molecular processes influencing the performance of the molecular recognition peptide will be expanded and discussed.

#### 2.2.1. Non-Specific Peptide-Surface Interactions Influence Bioreceptor Performance

**Figure 5:**
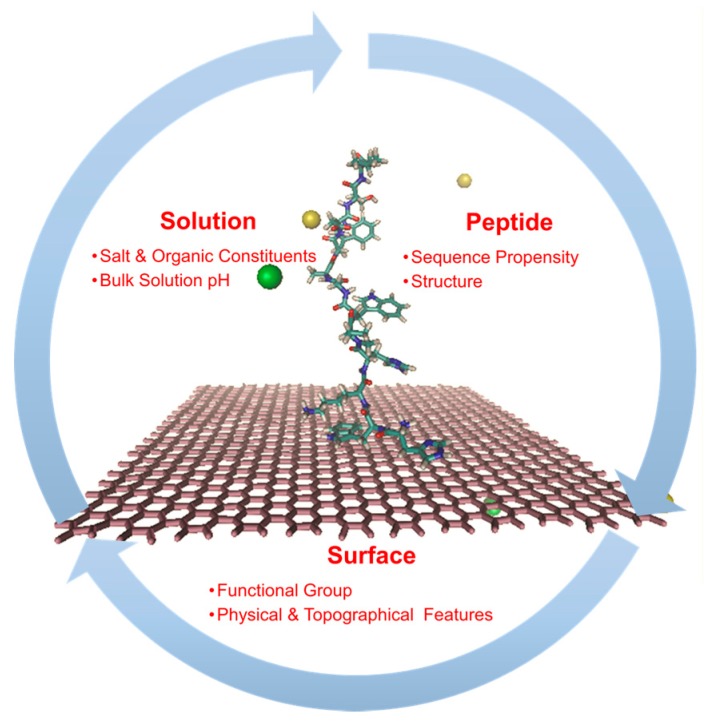
Factors influencing the non-specific interaction between the peptide and surface. The peptide adsorption onto the graphene layer is shown in the center of the process cycle. Water molecules are not explicitly shown for the ease of visualization. The sodium ions are shown in yellow and the chloride ions are shown in green.

Within aqueous solutions, peptides tend to rapidly make and break H-bonds with nearby water molecules, resulting in a peptide’s dynamic nature [62,63,64,65]. The innermost of these water molecules comprise a hydration shell that is made up of a highly dense and ordered water layer, and the exchange of water molecules within this layer with bulk water is controlled by the exposed functional groups on the peptide surface [66,67,68]. The successive layers of water molecules surrounding the peptide play a crucial role in the molecular recognition properties of the peptide [66,69]. However, in the event of direct interaction of the peptide with a material surface, non-specific interaction can affect the mycotoxin recognition properties of the peptide. On a broad level, the non-specific interactions between a peptide and material surface involve the interplay between three different components—peptide, ions and solvent, and the material surface (Figure 5). Non-specific interaction of the peptide with the material surface generally involves the displacement of some of the outer layers of water from the underlying sorbent as well as the peptide’s surface to the surrounding bulk aqueous solution [70,71,72]. The spatial orientation of the redistributed water is further limited by the distribution of surface charge and functional groups on the underlying surface [73]. For example, in the case of a hydrophobic surface there exists a ‘hydrophobic gap’ immediately above the material surface that is otherwise adjacent to an ordered structure of water due to the inability of water to form H-bonds with the functional groups of the surface [74,75]. Alternatively, in the case of charged hydrophilic substrates, the interfacial water structure is influenced by the overall charge density on the surface which in turn is directly affected by the bulk solution pH [75,76,77,78,79,80,81]. As the surface charge density of a material surface varies with the bulk pH, counter ions in solution are attracted to the material surface while the co-ions are repelled. This results in localized charge accumulation at the material interface, which results in establishing a pH gradient between the material and bulk solution, which, in turn, affects the extent of ionization within the peptides. The charge density determined for the same material surface can however vary, for otherwise same conditions, depending on the type of cationic and anionic constituents within the solution. Smaller anions that have lower surface charge densities are known to interact strongly with the polar groups of the material surface [82], while larger ions that are singly charged, bind to material surfaces not only based on the charge but also due to van der Waals forces [83]. For example, non-specific interactions between the material and peptide are promoted on a hydrophilic surface in the presence of kosmotropic ions (e.g., PO_4_^3−^, SO_4_^2−^, Al^3+^, Mg^2+^) as opposed to chaotropic ions (ClO_4_^−^, NO_3_^−^, NH_4_^+^, N(CH_3_)^4+^) since the entropic benefits associated with the release of ordered water is more favorable with the former type as opposed to the latter type of ions. In addition to ionic solutes, non-ionic solutes are also important constituents in solution that could perturb the water structure surrounding functional groups on material surface as well as peptides [70,84]. While solute-induced effects on a native peptide’s structure is well documented, its effects on the immobilized peptides are yet to be adequately understood [81,83,84,85,86,87,88,89,90]. Consequently, the interaction of a charged hydrophilic surface with an aqueous solvent can vary between very weak to very strong material-solvent H-bond interactions.

Depending on the material in an aqueous environment, a folded peptide structure can be expected to exhibit different affinities to the material surface in different regions of its 3-D structure. Thus, it can be expected that on hydrophilic interfaces, peptides predominantly expose those patches that are rich in hydrophilic residues toward the material surface and on hydrophobic surfaces, peptides would direct their hydrophilic residues elsewhere, leaving their hydrophobic patches to the material surface. Similarly, peptides on positively or negatively charged surfaces tend to orient themselves to expose oppositely charged regions to the surface. Furthermore, the peptide can be expected to preferentially orient on a strongly interacting surface while it can be expected to exhibit random orientations on weakly interacting material surface. For this purpose, many of the earlier studies were carried out to understand the affinity of specific amino-acid groups with the functional groups on the material surfaces. The goal of which was to better predict the strength of non-specific interaction between the peptide receptors containing different primary sequence and diverse exposed functional groups on the material’s surface. However, different homopeptide studies and experimental binding studies on tandem repeat peptide sequences have demonstrated that the interfacial binding strength of a given sequence is not an additive sum of the binding strengths of the individual residues [91], but is instead influenced by the structure and order of the peptide sequences [55,78]. The strength of H-bond interactions between the material-solvent further determines the extent of desolvation of the peptide. Such spatial restructuring of water, along with potential loss in hydration sheath from the peptide and material surface, together promote the non-specific interactions between the peptide and the surface, influence the peptide’s orientation on the material’s surface, reduce the peptide ‘*fluidity*’, and alter the peptide’s native conformational preferences [77,78,92,93]. The configurational preference of the immobilized peptide could in turn determine the *performance* of the molecular recognition receptor.

In addition to the type of functional groups presented by the material surface, physical properties and topographical features of the material surface and the type of solvent environment can also have profound effect on the peptide-surface interactions [54,91,94,95]. In the interpretation of many peptide-surface interaction experiments, it is often assumed that the surface structure of the material is stable, which is not always the case, as commonly observed in glasses, which can undergo surface dissolution and ion release from the surface) and polymers, which can undergo structural rearrangements or hydrolysis (if hydrolytically degradable) [96]. For example, in atomic force microscopy experiments of a model peptide on Nylon 6/6, the effective standard-state adsorption free energy values deviated markedly from values obtained on a model surfaces with a similar functional group chemistry (-NHCOCH_3_) [97]. The authors attributed the deviation in measured standard-state adsorption free energy values to the swelling behavior of Nylon surface as such structural rearrangements of long chains in polymers are not uncommon in the field of biomaterials and other areas of material science. Other topological features of the surface such as its curvature, electrical-electronic nature, crystalline orientation, and molecular architecture have been known to contribute to non-specific interaction and may influence the molecular recognition properties of the peptide bioreceptor [98,99,100,101]. Similarly, as stated before, the working environment of peptide-based biotransducers for OTA monitoring involve methanol or ethanol additives (e.g., wine), that are less polar than water and tend to form linear H-bond with the peptide. Peptide interactions with anhydrous organic solvents have been known to weaken the internal stability of the peptide, and could render the peptide inactive [102]. However, studies have also indicated that peptides might perform better (increased affinity and enhanced selectivity) in mixed solvent matrices of appropriate composition [102,103]. This area is becoming increasingly more tractable using explicit co-solvent models.

Clearly, the impact of non-specific peptide-surface interactions on the molecular recognition of a sensing peptide receptor is non-trivial and requires careful characterization of the multiple factors contributing towards such non-specific peptide-surface interactions. The fundamental problem of one-variable-at-a-time immobilization procedures is that it is laborious, time-consuming, expensive, and even when accompanied by multi-factorial approaches; the likelihood of such an approach leading to a design strategy with optimal sensor performance is extremely small. Appropriate engineering control over the performance of the peptide-based sensing element will however, require a thorough assessment on the impact of these design parameters on the in solution mycotoxin binding properties of the peptide-based molecular receptor.

#### 2.2.2. Site-Specificity and Type of Chemical/Biological Modification Influence Sensing Element Performance

**Figure 6:**
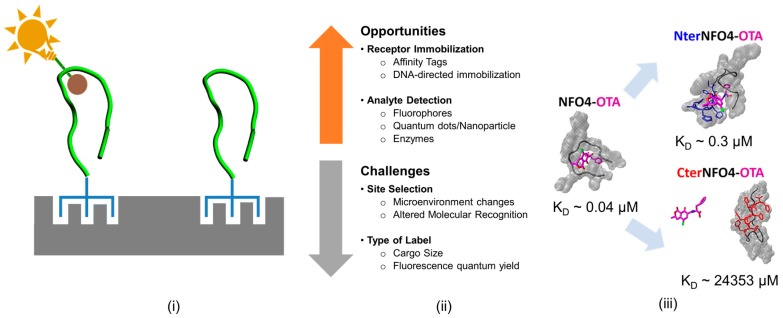
Chemical/biological modification in biosensor design. (**i**) Schematic of the peptide molecular recognition receptor immobilized on the biotransducer; (**ii**) Challenges and opportunities for site-specific modification; and (**iii**) Differences in the in-solution OTA binding affinity of NFO4 when 6× His-tags are placed on the N-terminus (NterNFO4-OTA) and C-terminus of NFO4 (CterNFO4-OTA). Reproduced from [29], Copyright ACS publications, 2017.

Chemical/biological modification of sensing element involve stoichiometric alteration of a single or unique amino acid residue either via a biological moiety, chemical agents, fluorophore or a radiolabel, and serve one or both of the two main purposes—(a) peptide immobilization via attachment of linker molecules to the functional groups on the sorbent surface and (b) analyte detection via shifts in optical properties of fluorophore [57,104,105]. Figure 6 summarizes the opportunities and challenges in chemical/biological modification of peptides.

Linkers can be appended to the peptide receptors by site-selective modification of the amino acid via chemical modification or by synthesis of fusion peptides in expression vectors such as *E. coli* ER2738 [57,104,105]. Linkers bind with high specificity to a target biological or a chemical ligand on the adsorbent phase. In so doing the linkers facilitates the immobilization of the molecular recognition entity on the detection platform while optimally orienting it toward the solution phase for hapten capture. Several strategies have been adopted in the literature for immobilizing peptides via linkers including: (1) biotinylation of peptide receptors and their subsequent immobilization via streptavidin-coated supports; (2) DNA-directed immobilization; and (3) affinity tags (e.g., hexahistidine tag (6× His-tag)). While ideally the direct interactions of linker with the peptide receptors are not desired, such interactions are not entirely avoidable. As a result, the introduction of linkers could have either positive or negative effects on the biochemical properties of a peptide receptors. In addition to the type of linker, the site of linker placement can also affect the intrinsic biochemical activity of the molecular recognition entity of a sensing element. For example, drugs conjugated to antibodies via glutamine tags showed differences in their pharmacokinetics depending on the location of the drug on the heavy and light chains of the antibodies [106]. Similarly, on solid platforms, NFO4 oriented with 6× His-tag on the N-terminus was found to be significantly better at hapten recognition than those oriented with the affinity tags on the C-terminus of the peptide [29,32].

Though many of the recent approaches have relied on label-free approaches to detect peptide-mycotoxin binding, chemical/biological modification of sensing element to incorporate reporter tags within the sensing element are still popular. Many of the widely applied reporter tags rely on the fluorescence and chemiluminescent properties of the tag. Some of the commonly used fluorescent derivatives include amine-reactive isothiocyanate of fluorescein and rhodamine, amine-reactive succinimidyl esters such as NHS-fluorescein, and sulfhydryl-reactive maleimide activated fluorophores such as fluorescein-5-maleimide. Assays relying on the oxidizing nature of the enzyme horseradish peroxidase to generate chemiluminescent signal have also been widely applied. In addition to chemical derivatization, fluorescent-tags could also be expressed via genetic encoding or via nanoparticles. The reporter tag helps visualize and quantify peptide-mycotoxin binding by tracking the changes in its spectral properties such as luminescence or polarity. The kinetics and duration of fluorescence or its reversibility will then provide information on the affinity and dynamics of the peptide binding to mycotoxin. The robustness of such response is however, directly associated with the photochemical and photo-physical characteristics of the fluorophore which in turn is dependent on the brightness, and photo-stability of the probe, as well as its sensitivity to changes in the environment. Recent studies have also demonstrated that the placement of fluorophores such as AlexaFluor and Ni-NTA-Atto in immediate vicinity of the peptides with the histidine tags improves the fluorescence quantum yield and facilitate high-resolution imaging [107,108]. Other modifications such as phosphorylation of the tyrosine residue, chelation of the fluorophore, and certain types of nanomaterials were all found to improve the optical performance of hapten monitoring sensor. In contrast, canonical amino acids like tryptophan and tyrosine or nanomaterials with similar molecular structure can quench the overall fluorescence of organic fluorophores through intramolecular π-π stacking interactions, thereby reducing their fluorescence quantum yield. Another challenge in the fluorescent tagging process pertains to the cargo size of the tag. Ideally, tags are not expected to affect the molecular recognition events and the peptide should be able to recognize its target with a high level of specificity and selectivity. However, appending enzyme molecules such as horseradish peroxidase that are bigger than (>3× molecular size) the size of either the peptide or hapten could affect the binding dynamics and could potentially influence the molecular recognition process in ways that are difficult to anticipate. In this regard, the challenge for the field now is to devise conceptually new strategies for chemically modifying peptides with smaller labels that provides a robust and reliable response as a function of the type of sensing platforms.

From the above discussions, it is, therefore, evident to the readers that multiple factors are involved in the design and fabrication of the sensing elements, and the challenges involved in inching towards an optimal sensor performance is astronomical. Currently, the impact of non-specific peptide-surface interactions and chemical/biological modification on the mycotoxin recognition properties of a synthetic peptide is verified by cyclic experimental testing using routine biophysical techniques, such as equilibration dialysis, ELISA or SPR [44], all of which, as stated before, are inefficient in guaranteeing an optimal sensor performance. Therefore, the grand challenge in the fabrication of any sensing element lies in the ability to predict and control the performance of the molecular recognition receptors. It is in this context that MMS tools such as all-atom molecular dynamics (MD) are highly relevant in providing the molecular-level insights that is critical to fine-tune the bioreceptor’s performance.

## 3. Molecular Modeling Approaches towards Design and Fabrication of Peptide Bioreceptors with Optimal Hapten Binding

In this section, we explore the important considerations in the development and evolution of approaches to enable molecular level modelling of peptide receptors and their recognition of ochratoxins.

### 3.1. Basic Considerations in the Application of Molecular Dynamics Simulations to Peptide Bioreceptors

**Figure 7:**
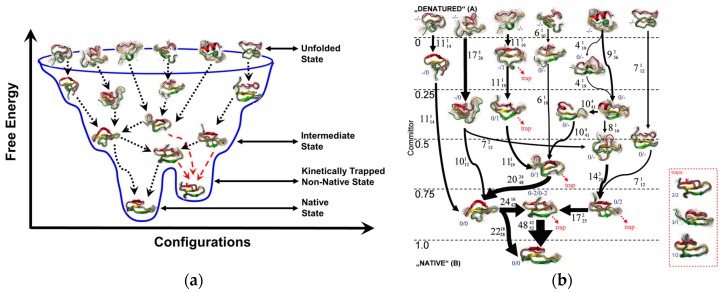
Peptide conformational ensemble. (**a**) Simplified view of the folding funnel for peptide domain, PinWW; (**b**) Detailed outline of the flux and pathway for the peptide folding. The local energy minima traps are shown in red (dotted lines) and the global energy pathway is shown in black (dotted and solid lines). Reproduced from [116], Copyright PNAS 2009.

Peptide bioreceptors and mycotoxins together comprise hundreds of atoms with hundreds of rotatable bonds. The number of rotatable bonds is in turn responsible for the exponential rise in the diversity of peptide and mycotoxin conformations. At equilibrium, peptides exist in an ensemble of conformational states, each with their respective probabilities (Figure 7). Within this ensemble of peptide conformations, mycotoxins bind with high affinity to those conformations that produce the bound state of the peptide, while weakly interacting mycotoxin-peptide pairs are associated with other conformational states of the peptide. The binding of the mycotoxin with the peptide may also be accompanied by a change in shape of the reacting molecules and their proximal water structure. It is important to note that the binding process may not necessarily involve a single conformation but in fact a smearing of energetically similar conformations, or potentially, an alternate minimum. Consequently, mycotoxins that bind to the peptides with high affinity tend to shift the population distribution to favor the bound states of the peptide-mycotoxin complex; and mycotoxins that bind to the peptides with low affinity tend to skew the equilibrium toward the unbound states [109,110,111]. The foregoing requires that a clear energetic distinction be established between bound and unbound states, for which energetically accessible receptor and mycotoxin conformations should be calculated. For such type of molecular estimations and visualization, all-atom MD simulations are appropriate and quite frequently used [28,29,112,113,114,115].

MD methods are appropriate for characterizing the sub-molecular processes involved in bioreceptor performance [54,117,118]. All-atom MD methods empirically set parameters to represent the interactions between the atoms contained in a given molecular system and simulate the dynamic properties of the system by solving Newton’s equations of motion (*aka* force field) [117]. A force field equation calculates the total potential energy of a molecular system by summing up contributions to the potential energy from the various types of atom-atom interactions in the system [54,117,118]. The force vectors acting on the atoms are then obtained when the potential energy acting on the atom is differentiated with respect to atomic position [118]. These state-of-the-art techniques are capable of providing a real-time scenario of the molecular-level changes involved in the structure, dynamics, and thermodynamics of the peptide bioreceptor following the application of known biophysical principles. However, the use of the above-described regular MD approaches to simulate the conformational behavior of the peptide-mycotoxin binding process is computationally very inefficient. Inefficiencies arise because of the extremely large number of degrees of freedom that must be represented in a simulation and because of the inherent problem of very slow phase-space sampling that arises due to the presence of relatively high energy barriers separating the extremely numerous local energy minima separating the conformational states contained within these types of systems. The challenge of extensive conformational sampling is made more acute by the need to use an explicit description of liquid water. An explicit description of liquid water is necessary because spatial restructuring of interfacial water (solvent) is a key determinant in the peptide interaction to a biotransducer’s material surface and to the mycotoxin binding process. Therefore, adequate but efficient sampling of the conformational space or the free energy landscape is indispensable for ensuring meaningful results. Efficient methods of searching the conformational phase space or conformational sampling of a complex molecular system have been developed and previously reviewed [117,118,119,120,121].

Our group has focused on the use of a conformational sampling approach involving time dependent bias-potential [*aka* biased exchange metadynamics (BEMD)] for the purpose of modeling peptide bioreceptor design and synthesis [28,29]. BEMD involves conformational sampling at the same temperature but explores the conformational landscapes of the biomolecule by introducing a time-dependent bias potential as a function of the chosen collective variables (CV) [122,123,124,125]. The CVs are explicit functions that control the atomic coordinates of the biomolecule and describe the slow-order transition from its initial conformation to metastable conformation [122]. Each replica in a BEMD simulation is biased with a time-dependent bias-potential acting on one or two different CVs. After a certain time, exchanges between different pairs of replicas are attempted using the Metropolis scheme. If the exchange is accepted, the CV of replicas involved will be also exchanged. Thus, the exploration of the conformational landscape that was initially biased in the direction of the first CV continues its exploration biased by the second CV (and vice versa). In this manner, many different conformations can be simultaneously explored by BEMD simulation as opposed to a regular MD simulation. Nevertheless, the time in any conformational sampling simulation does not represent the actual duration of continuous dynamics but rather the amount of sampling that was conducted. In other words, while the sampling approaches determine the ensemble average of states, the trajectories do not necessarily represent the actual dynamic time sequence of events and therefore limits any direct kinetic estimates of the biomolecular events from such simulations. In such a scenario, predictive analytics such as Markov State Model (MSM) can greatly aid the prediction of dynamic properties involved in peptide–mycotoxin binding, e.g., binding free energy, dissociation constant, and ‘*on*’ and ‘*off*’ rates [28,29]. MSM approaches involve the linear transformation of the configuration space into collective coordinates that are then sorted by ‘*slowness*’ into discrete states, such that the transitions within each discrete state are fast but the transitions across different states are slow. The transition probabilities between each of the states would then provide a kinetic estimate of these transitions and provide insights into the affinity of the binding pair and selectivity of a peptide bioreceptor. In this regard, the utility of combined BEMD and MSM approaches to the design of peptide bioreceptors for biotransducers is listed below.

### 3.2. Evaluating the In-Solution Mycotoxin Recognition Properties of Peptide Bioreceptors

Measured binding affinities of peptides to mycotoxins are experimentally obtained under environmental conditions that are unique to the study design. In such studies, the *in-solution* binding dynamics could be potentially compromised by routine experimental practices such as peptide conjugation, bio-immobilization, or ochratoxin tagging, such as by conjugation with a larger reporter enzyme. These practices are essential for the physicochemical detection and monitoring of the binding reaction. In a recent MD study, the author evaluated the affinity of the first four peptides listed in Table 2 i.e., (a) hexamer (SNLHPK); (b) octamer (CSIVEDGK); (c) NFO4 (VYMNRKYYKCCK); and (d) 13-mer (GPAGIDGPAGIRC) to OTA and its non-chlorinated analogue, OTB, in a wine-like solution environment (pH = 4.0) [28]. Experimental studies had previously documented the affinity of each of these peptides to OTA [39,46,47]. Although the affinity data was not available for the majority of these peptides, the author and co-workers had experimentally determined the selectivity of NFO4 peptide to OTA in the presence of OTB [32,47]. The MD approach involving BEMD and MSM qualitatively reproduced the affinity ranking for the first four peptides to OTA in Table 2. More importantly, the MD studies demonstrated that all the peptides except for hexamer, interacted strongly with the ester (R_1_COOR_2_), amide (R_1_CONHR_2_R) and phenol segments of the ochratoxins. Despite such stronger non-bonded interactions, the superior affinity and selectivity of NFO4 stemmed from the lower solvation penalties associated with the NFO4-OTA complex as opposed to other peptide-mycotoxin combinations. Moreover, a key residue directly responsible for the higher selectivity of NFO4 to OTA as opposed to OTB was the solvation penalty differences in the lysine residue at the 12th position of the peptide. It was speculated that a point mutation or the elimination of charge by some modification of Lys-12 might likely reduce or eliminate the OTA selectivity of NFO4.

As the study demonstrated, the application of standardized conditions via the combined BEMD and MSM approach, provided a uniform platform to compare the mycotoxin’s recognition properties of different peptides and identify the key energetic component influencing the peptide affinity and selectivity, that were otherwise not obtainable using routine biophysical techniques or regular MD approaches. Future studies will seek to include more peptides from Table 2 to match the experimental results and the ranking of mycotoxin recognition efficiencies of differently structured peptides. Subsequent ranking of these structures would provide molecular insights critical to identifying diverse amino acid blocks or key residues with varying affinity to different zones of a target mycotoxin. As many mycotoxins share similar chemistries and structural homology (Figure 1), the peptide motifs could subsequently serve as the initial template to mutate or add or delete amino acid for designing new peptides with improved molecular recognition properties against a specific class of mycotoxins.

### 3.3. Evaluating the Effect of Site-Specific Modifications on Recognition Properties of Peptide Bioreceptors

The authors have recently developed a reusable, combined fluorescence and electrochemical bioassay for ochratoxins by immobilizing NFO4 onto a microporous solid support and demonstrated high specificity and low detection limit [30,31,32]. The NFO4 was immobilized via 6× His tags onto a Zn^2+^ laden chitosan foam. Two modifications of the NFO4 was evaluated, namely; 6× His placed on the N-terminus (Nter-NFO4) and 6× His placed on the C-terminus (Cter-NFO4) of the NFO4, respectively. The Nter-NFO4 were found to be 3 orders of magnitude better at OTA recognition than the corresponding Cter-NFO4. However, as with much of such experimentation, correlations are apparent but causation is more elusive. For example, it was not clear from the above study if the observed site-specificity of the affinity tags arose because of the location of the tethering tag or because of interactions of the NFO4 peptide receptor with the chitosan foam pursuant to the immobilization. As an initial effort to delineate the confounding impact of chemical/biological modifications on the performance of a molecular recognition receptor, the authors assessed the impact of site-specific affinity tagging (6× His tag) on the *in-solution* binding behavior of NFO4, especially when the affinity tags are comparable in size and chemistry to the native peptide [29]. Ideally, the tags were expected to be passive in their interaction with the peptide and not to interfere with its molecular recognition properties. However, MMS studies suggested that the addition of affinity tags to particular ends of the NFO4 had significant impact on the folding and the binding behavior of the peptide. In general, the tagged peptides favored solvation more than the untagged peptide. While the 6× His tags placed on the N-terminus of the NFO4 (Nter-NFO4) generally preserved the native fold of the peptide, the tags altered the intrinsic contact network and actively interacted with OTA. Surprisingly, the Nter-NFO4 had an order of magnitude weaker affinity to OTA than the native NFO4. In contrast, the 6× His tags placed on the C-terminus of the NFO4 (Cter-NFO4) improved the stability of the native peptide by altering the native fold and the intrinsic contact network within the peptide. In fact, Cter-NFO4 was found to have five orders of magnitude weaker affinity to OTA than Nter-NFO4. The addition of tags did not affect the affinity of the peptide to OTB. Consequently, and consistent with experimentation, the Nter-NFO4 preserved the generally affinity and selectivity of NFO4 while the Cter-NFO4 eliminated the OTA-specific binding capabilities of NFO4.

Two key points are noteworthy. Firstly, N-tagging reduced the OTA affinity by one order of magnitude and C-tagging reduced the OTA affinity by five orders of magnitude. Recall, experimental discrimination resulted in three orders of magnitude difference. Secondly, the step-wise binding mechanisms of the NFO4 and Nter-NFO4 to OTA were quite different. The OTA-specific binding behavior of NFO4 was entirely driven by the lower solvation penalty of the NFO4-OTA complex, as opposed the OTA-specific binding by Nter-NFO4 that was driven by the interplay of non-bonded interactions and solvation penalty associated with the peptide-OTA complex. The simulated results were again in good qualitative agreement with the experimentally derived results [29,31,32].

As tags were not expected to actively influence the peptides’ mycotoxin binding properties, the above study clearly showed that the tags in fact influence the molecular recognition properties of the peptide and may not necessarily be passive in its interaction with the peptide. In this regard, MD platforms could be a valuable resource for screening chemical/biological tags and assessing its impact on the molecular recognition properties of the peptide receptor. Future experimental strategies may also be directed to explore and validate strategies that would minimize the direct interaction between the peptide and the tags by introducing more flexible spacers such as polyethylene glycol (PEG) of poly-l-lysine. Could such spacers, strategically placed between the NFO4 and the 6× His tag, recover the native binding properties of the NFO4 irrespective of the site of modification?

### 3.4. Evaluating the Effect of Interfaces with Solid Substrates on the Recognition Properties of Peptide Bioreceptors

While the ability of empirical force fields to accurately represent the molecular behavior of peptides within an aqueous phase is well documented, the peptide interactions with a biotransducer surface or its behavior within the interphase formed between the material and solvent may not necessarily be similar [117]. In one of our earlier studies, regular MD simulations was applied to assess the binding affinity of five discrete peptides with single walled carbon nanotube (SWCNT) and graphene in explicit water [112]. From this study, it was determined that the peptides were concertedly adsorbed and the adsorption behavior was governed by peptide segments. The curvature of the carbon-based nanomaterial was an additional factor contributing to the adsorptive interaction of peptides with the SWCNTs and thus two challenges need to be addressed. One of the primary challenges involved in modeling the peptide interaction at the solid interface is with regard to the direct application of classical force fields such as CHARMM [98,99,126,127,128,129], AMBER [112,130], and others [112,131]. Over the years, many types of classical force fields have been developed, each designed to give a certain balance of accuracy and speed to answer very specific questions [54,117,118]. Many groups have since developed a separate set of force field parameters to describe the behavior of the peptide at the solid-liquid interface [54,117,118]. Such parameters are currently available for a few model surfaces such as crystalline glass, high-density polyethylene, and graphene [99,114,115]. However, the interfacial force field parameters are limited to a few materials and need to be optimized for every newer generations of force fields. For example, the interfacial force field for graphene, GRAPPA, was optimized for charmm22 force field and cannot be directly ported to charmm36 force field [99]. In fact, a recent study suggests that charmm36 force field could be directly applied without any modification to simulate the adsorption behavior of small molecules on carbon-like nanomaterials [127]. Nevertheless, the reliable application of the newer generation of empirical force fields for design and fabrication of molecular recognition bioreceptors must be preceded by the preliminary validation of the force field by matched experimental data.

In addition to the challenge posed by the classical force fields, considerations must also be given to the data refinement and analysis methods when the standard data-reduction algorithms cannot be applied to peptides immobilized on a surface [113]. Rotational and translational freedom of the peptides in solution are not restrained in either of the three dimensions, and therefore, the traditional approach of using cluster analysis to group peptides together on the basis of structure rather than orientation is justified. However, in the case of an immobilized peptide, structure and orientation both play important roles in mycotoxin recognition. Two types of methods were recently developed to address this challenge [113]. In the first method, the sampled immobilized states were clustered based on their orientation and their distance from the surface. This ensures that peptides that are non-specifically interacting with the surface do not appear in the same cluster as those peptides that are not interacting with the surface. However, there are instances wherein this method is not suitable. For such cases, a second cluster method was developed wherein peptides were clustered together on the basis of conformation and orientation without regard to the distance from the immobilized plane. Our future MD studies, will seek to address these specific challenges, at least on carbon-based nanomaterials in tandem with BEMD and MSM approaches.

## 4. Conclusions and Outlook

Food-derived mycotoxins pose considerable health concerns and can have broad economic, social and environmental impacts, especially in low-income economies. Since minimizing mycotoxin exposure is the main therapeutic recommendation, producers and regulators are seeking affordable hapten-sensor systems. Such mycotoxin monitoring microsystems need to be supported by optimized performance with improved sensitivity, selectivity, and detection range in order to facilitate such a paradigm shift. To enable the practical, on-site implementation of food safety requirements, the biosensor systems also need to record as many potential mycotoxins as possible in parallel and within a short period. Multi-analyte detection systems that employ biotransducers of peptide arrays will therefore need to be developed. These peptides will need to be biomolecularly engineered to maximize specificity but also minimize cross-reactivity in a bid to eliminate false negatives. Moreover, these detections systems may be single or multi-modal and thus may accommodate multiple peptide-labelling approaches.

Our experiences have shown that traditional experimentation, especially one-variable-at-a-time approaches, may not be sufficient to address the performance challenges posed by peptide-based receptors for mycotoxin recognition. In addition, it is not always possible to capture experimentally such sequential molecular interactions. Instead, the incorporation of an MMS approach, such as an all-atom MD approach to screen and manipulate the sub-molecular interactions occurring on a biotransducer’s surface, might provide a more sustainable route to optimizing the performance of molecular recognition receptors. In this paper, the use of MD approaches in the development of peptide-based biotransducers was demonstrated to be relevant in three specific areas: (a) screening and optimization of peptide receptors with defined molecular recognition properties to a target mycotoxin; (b) screening and optimizing the type and placement of chemical or biological tags and labels on a peptide receptor; and (c) strategies to minimize the influence of non-specific peptide-surface interactions once immobilized. It is noteworthy that the capabilities of classical MD approaches to successfully simulate receptor performance is still evolving and must first be carefully developed and validated for specific applications. Our group’s approach has been to use classical force field in tandem with enhanced sampling (such as BEMD) to rapidly screen diverse peptide conformations relevant to mycotoxin binding and predictive analytics (such as MSM) to predict the kinetics of such conformational transitions. The utility of BEMD and MSM approaches to (a) improve the mycotoxin recognition properties of the peptide receptor and (b) to assess the impact of site-specific affinity tags on the mycotoxin recognition properties of the peptide receptor have been demonstrated, and its potential impact on biotransducer fabrication has been outlined. The utility of BEMD and MSM approaches on discontinuous phases is, however, yet to be tested. However, with the appropriate choice of force field that accurately represents the molecular behavior of molecular recognition receptors and validation with experimentally measurable behavior, MMS tools will continue to grow as a critically valuable resource for biosensing applications. Once appropriately tested and validated, the integration of MMS tools to mainstream biosensor design and fabrication will minimize the product development lifecycle and provide a rational approach to tweak the device performance. Furthermore, such a predictive framework will also facilitate the parallel detection of multiple mycotoxins. The better use of resources, faster turnover of sensor systems with improved performance, and the parallel detection of multiple mycotoxins would all translate the peptide-based molecular recognition receptor as a more affordable alternative to the more expensive antibody-based molecular recognition receptors currently used to monitor the mycotoxin content in food/feed. The widespread availability of affordable biosensors would eventually empower food producers with self-regulation and support access to a wider range of export markets with diverse regulatory requirements. These developments will in the longer run help to reduce food loss and reduce global hunger, strengthen local economies while safeguarding human health.

## Figures and Tables

**Table 1:** Performance comparison of the different receptors currently used in OTA monitoring.

Recognition Molecule	Affinity (μM)	Selectivity
Human serum albumin ^1^	0.019–1	Not selective
Antibody ^2^	0.00001–0.083	20
DNA Aptamer ^3^	0.096–0.370	6–100
Peptide ^4^	0.00007–29.4	3

^1^ See references [6,39]. ^2^ See references [37,40,41]. Estimate from immobilized system. 20 fold selective to OTA when immobilized. ^3^ See references [34,42,43]. Solution-based estimate. 6–100-fold selectivity to OTA. ^4^ See references [14,39,44,45,46]. Estimates based on standard solid-phase and solution-based assay. The OTA selectivity was experimentally derived and is only available for reference [47].

**Table 2:** Performance of the peptide receptors currently used for OTA monitoring, arranged in an increasing order of its affinity to OTA.

Petpide Sequence	Screening Approach	Affinity (μM)-Expt ^1^	Affinity (μM)-Predicted ^2^	Reference
SNLHPK	Phage display library	29.4	1991	[46]
CSIVEDGK	Molecular modeling	12.0	1861	[39]
GPAGIDGPAGIRC	Molecular modeling	16.0	2563	[39]
VYMNRKYYKCCK	Mimotype mapping—NADH-FMN oxidoreductase	0.079	1.47	[47]
IRPMVDP	Mimotype mapping—mAb	0.0017	NA	[49]
AETYGFQLHAMK	Mimotype mapping—2nd gen peptide library	0.00013	NA	[50]
GMVQTIF with pentamer spacer (GGGSK) and biotin tag	Mimotype mapping—mAb 2A11	0.00007	NA	[44]

^1^ Affinity values were obtained from the reported ELISA standard curves at half inhibition and should be treated as a generic representation of the peptide’s affinity to OTA and may not be necessarily accurate. Experimental variables unique to each study can influence the accuracy of the affinity estimates. ^2^ The peptide’s affinity to OTA (*K_D_*) was predicted using the equation $K_{D}=\frac{1}{\exp\left(-\frac{\Delta G}{RT}\right)}$ where *R* = 0.00831 kJ·mol^−1^·K^−1^, *T* = 298 K and Δ*G* is the computed free energy involved in the peptide-OTA binding. NA refers to the affinity estimates that which were not computed. The peptide, SNLHPK, was predicted to be not selective to OTB. The peptides, CSIVEDGK and GPAGIDGPAGIRC were predicted to be 2 times more selective to OTA than OTB. VYMNRKYYKCCK was predicted to be 13 times more selective to OTA than OTB.
